# Danaparoid sodium inhibits systemic inflammation and prevents endotoxin-induced acute lung injury in rats

**DOI:** 10.1186/cc6851

**Published:** 2008-04-02

**Authors:** Satoshi Hagiwara, Hideo Iwasaka, Seigo Hidaka, Sohei Hishiyama, Takayuki Noguchi

**Affiliations:** 1 Department of Brain and Nerve Science, Anesthesiology, Oita University Faculty of Medicine, Oita, Japan

## Abstract

**Introduction:**

Systemic inflammatory mediators, including high mobility group box 1 (HMGB1), play an important role in the development of sepsis. Anticoagulants, such as danaparoid sodium (DA), may be able to inhibit sepsis-induced inflammation, but the mechanism of action is not well understood. We hypothesised that DA would act as an inhibitor of systemic inflammation and prevent endotoxin-induced acute lung injury in a rat model.

**Methods:**

We used male Wistar rats. Animals in the intervention arm received a bolus of 50 U/kg of DA or saline injected into the tail vein after lipopolysaccharide (LPS) administration. We measured cytokine (tumour necrosis factor (TNF)α, interleukin (IL)-6 and IL-10) and HMGB1 levels in serum and lung tissue at regular intervals for 12 h following LPS injection. The mouse macrophage cell line RAW 264.7 was assessed following stimulation with LPS alone or concurrently with DA with identification of HMGB1 and other cytokines in the supernatant.

**Results:**

Survival was significantly higher and lung histopathology significantly improved among the DA (50 U/kg) animals compared to the control rats. The serum and lung HMGB1 levels were lower over time among DA-treated animals. In the *in vitro *study, administration of DA was associated with decreased production of HMGB1. In the cell signalling studies, DA administration inhibited the phosphorylation of IκB.

**Conclusion:**

DA decreases cytokine and HMGB1 levels during LPS-induced inflammation. As a result, DA ameliorated lung pathology and reduces mortality in endotoxin-induced systemic inflammation in a rat model. This effect may be mediated through the inhibition of cytokines and HMGB1.

## Introduction

Despite extensive investigation of strategies for treating acute lung injury (ALI), the overall mortality still remains high at approximately 30 to 50% [1]. One of the mechanisms of sepsis-induced acute lung injury involves bacterial endotoxin release into the circulation that activates interconnected inflammatory cascades in the lung, ultimately leading to lung damage [2,3]. The production of inflammatory mediators plays an important role in the pathophysiology of inflammation in lung injury.

High mobility group box 1 (HMGB1) protein is an intranuclear protein that was originally identified as a DNA-binding protein, [4], but is also a late-phase mediator in the pathogenesis of sepsis [5]. HMGB1 acts as a pro-coagulant [6], thereby enhancing the inflammatory response in septic shock [7,8]. The timing of its release and action is typically later than other cytokines, such as TNFα and IL-1β [5]. Inhibitors of HMGB1 might therefore be beneficial in the treatment of various inflammatory diseases.

The role of clotting factors as inflammatory mediators has attracted close attention. Initiation of the coagulation cascade and the subsequent production of proinflammatory cytokines (particularly in response to factor Xa (FXa)) are central to the pathogenesis of sepsis [9,10]. Danaparoid sodium (DA) is a low molecular weight heparinoid consisting of heparan sulfate, dermatan sulfate and chondroitin sulfate that has both anticoagulant and anti-inflammatory effects. DA inhibits of FXa and factor IIa (FIIa) at ratios greater than heparin, while enacting minimal effects on platelet function [11-13]. Anti-inflammatory and anticoagulant agents have thus become a focus of new treatments for sepsis [14,15].

We hypothesised that DA would act as an inhibitor of systemic inflammation and prevent acute lung injury in a rat model. To test this hypothesis, we investigated the impact of DA administration on serum and lung levels of HMGB1, serum cytokine levels and on lung histopathology in rats with lipopolysaccharide (LPS)-induced systemic inflammation. To further elucidate the mechanism of action of these effects, we assessed the impact of DA on HMGB1 and cytokine secretion by RAW264.7 cells.

## Materials and methods

### *In vivo *study

#### Materials

Danaparoid sodium was purchased from Organon Co. Ltd. (CC, Oss, The Netherlands). Lipopolysaccharide (LPS, O127:B8) was obtained from Sigma (St Louis, MO, USA). Antibodies to rabbit polyclonal IgE anti-HMGB1 were purchased from Becton Dickinson and Company (Franklin Lakes, NJ, USA). Antibodies to β-actin were obtained from Abcam PLC (Cambridge, UK).

#### Treatment protocol

The study was approved by the Ethical Committee of Animal Research at the College of Medicine, Oita University, Oita, Japan. Male Wistar rats weighing 250 to 300 g (Kyudou, Saga, Japan) were used. Anaesthesia was induced by 4% sevoflurane. The animals were randomly assigned to one of three groups: (1) untreated LPS group: rats received a bolus of a 0.9% NaCl solution (1.0 ml/kg) and LPS (7.5 mg/kg) into the tail vein; (2) DA-treated LPS group: rats received a bolus of DA (50 U/kg), and LPS (7.5 mg/kg) into the tail vein; (3) Negative control group: rats received a bolus of 0.9% NaCl solution (1.0 ml/kg) into the tail vein. Before and after surgery, animals had unlimited access to food and water.

#### Histological analysis

A pathologist blind to group assignment analysed the samples and determined levels of lung injury according to Murakami's technique [16]. Briefly, 24 areas in the lung parenchyma were graded on a scale of 0 to 4 (0, absent and appears normal; 1, light; 2, moderate; 3, strong; 4, intense) for congestion, oedema, infiltration of inflammatory cells, and haemorrhaging.

#### Measurements of cytokine and HMGB1 secretion

HMGB1, Il-6 and TNFα levels were determined using a commercial enzyme-linked immunosorbent assay kit. HMGB1 was from Shino-Test Corporation, Tokyo, Japan; IL-6, IL-10 and TNFα were from R&D Systems Inc, Minneapolis, MN, USA.

#### Western blotting

Proteins were subjected to SDS-PAGE, and then transferred to polyvinylidene difluoride (PVDF) membranes (Millipore, Bedford, MA, USA). The membranes were incubated with primary antibody (1:1,000 dilution). After incubation with secondary antibody, blots were developed using an enhanced chemiluminescence detection kit (Amersham, Buckinghamshire, UK) and exposed on Hyperfilm ECL (Amersham). We used the NIH ImageJ software (National Institutes of Health, Bethesda, MD, USA) to quantitate protein band concentrations.

### Cell culture study

The murine macrophage cell line, RAW264.7, was maintained in RPMI 1640 medium containing 5% heat-inactivated foetal bovine calf serum and antibiotics at 37°C under 5% CO_2_. The medium was removed and replaced with RPMI 1640 containing 5% fetal bovine serum (FBS) for most experiments, or Opti-MEM (Sigma) for experiments designed to measure HMGB1 in conditioned media.

### Nuclear factor (NF)-κB binding assay

The DNA binding activity of NF-κB (p50/p65) was determined using an ELISA-based non-radioactive NF-κB p50/p65 transcription factor assay kit (Chemicon, Temecula, CA).

### Statistical analysis

For descriptive purposes, all continuous data were presented as mean ± SD. The data were analysed by Mann-Whitney U test for comparison between two independent groups. A p value of less than 0.05 was considered to be statistically significant. Survival data were analysed with the Kaplan-Meier program included in the Prism 4.0 software package (San Diego, CA, USA). p Values less than 0.05 were considered statistically significant.

## Results

### *In vivo *study

#### Mortality

**Figure 1:**
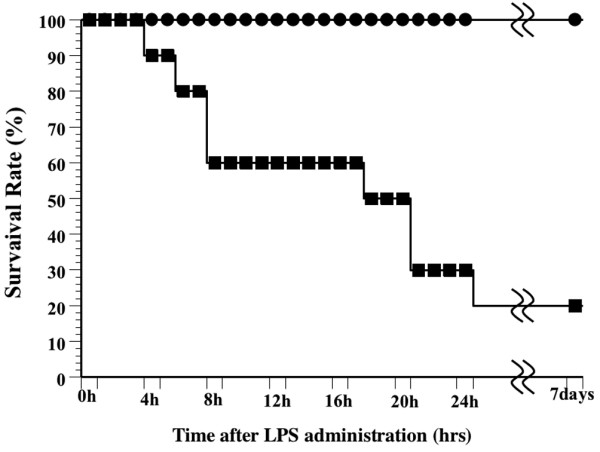
Effect of danaparoid sodium (DA) on the survival rate of lipopolysaccharide (LPS)-treated rats. The survival rate of animals treated with a bolus of LPS (7.5 mg/kg) into the tail vein (LPS group, n = 10) is represented by black squares. The survival rate of animals that received DA (50 U/kg) in addition to the intravenous injection of LPS (7.5 mg/kg) into the tail vein (DA treated LPS groups, n = 10) is represented by black circles.

A total of 40% of the rats in the untreated LPS group died within 12 h, and an additional 30% died within 24 h, while all rats in the DA-treated LPS group (50 U/kg) survived (Figure 1). In addition, only 20% of rats treated with 1 U/kg DA and 50% of rats treated with 10 U/kg DA survived for 24 h, suggesting a dose-dependent effect of DA on the survival rate of LPS-treated rats (data not shown). All of the saline-treated control animals survived for 7 days. Kaplan-Meier analysis revealed a significantly shorter time-to-death among the untreated LPS group compared to the DA (50 U/kg)-treated LPS group (p < 0.05).

#### Effect of DA on lung tissue specimens

**Figure 2:**
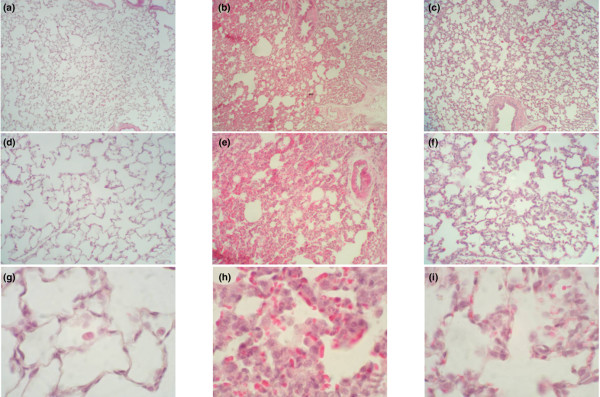
Effects of danaparoid sodium (DA) on lung histopathology in lipopolysaccharide (LPS)-administered rats. Rats were intravenously infused with either saline (control group), 7.5 mg/kg LPS (LPS group), or 7.5 mg/kg LPS with 50 U/kg DA (DA+LPS group). Lung tissue specimens were obtained from the negative control **(a) **magnification ×40, **(d) **magnification ×100, **(g) **magnification ×400); LPS **(b) **magnification ×40, **(e) **magnification ×100, **(h) **magnification ×400; and DA+LPS **(c) **magnification ×40, **(f) **magnification ×100, **(i) **magnification ×400 groups, respectively. Haematoxylin and eosin staining was used.

**Figure 3:**
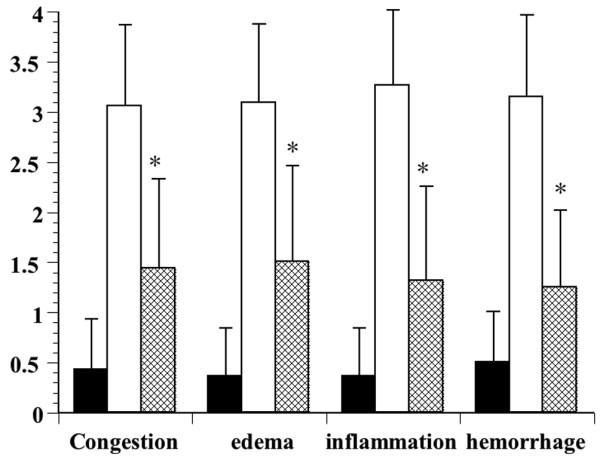
Effects of danaparoid sodium (DA) on lung histopathology score in lipopolysaccharide (LPS)-administered rats. The histological changes identified included congestion, oedema, inflammation, and haemorrhaging 12 h after the administration of LPS. White bars represent the non-injected control animals, black bars represent the animals injected with LPS, and slashed bars represent animals injected with DA and LPS. The data are expressed as the mean ± SD. *Denotes a significant difference compared with the LPS group (p < 0.05).

In the negative control group, no histological alterations were observed (Figure 2a,d,g). Among the LPS group with sepsis, the microscopic changes in the lung tissue specimens observed 12 h after LPS administration showed oedema-like formation, and interstitial infiltration by neutrophils (Figure 2b,e,h). The interstitial oedema and inflammatory cell infiltration were markedly reduced in the DA-treated group; DA treatment reduced each of these parameters. All of the scores were significantly lower in the DA (50 U/kg) group than in the LPS group (p < 0.05) (Figure 3).

#### Effects of DA on the serum levels of IL-6, TNFα, IL-10 and HMGB1

**Figure 4:**
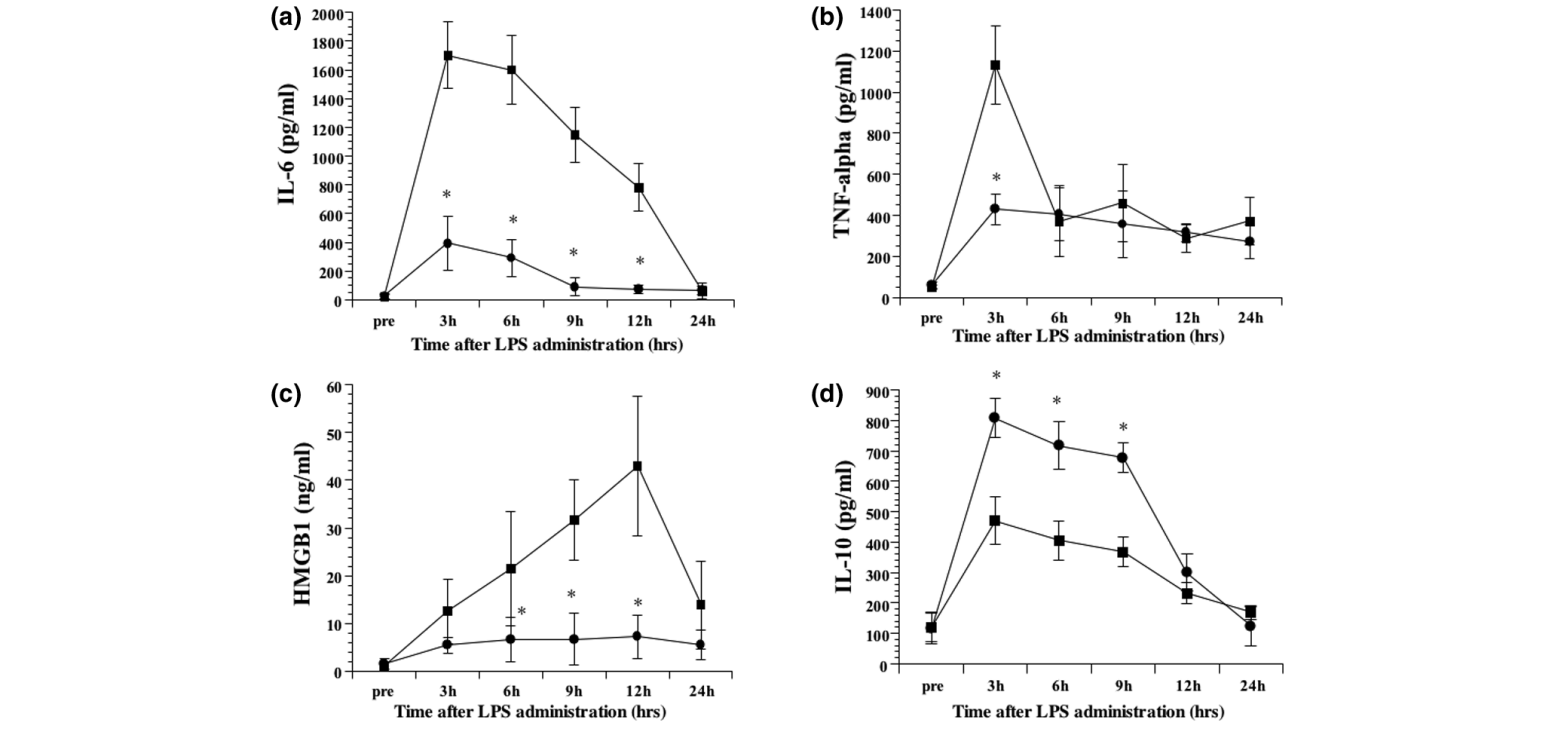
Temporal changes in the tumour necrosis factor (TNF)α, interleukin (IL)-6, IL-10, and high mobility group box 1 (HMGB1) serum concentrations following LPS administration. The IL-6 **(a)**, TNFα **(b)**, HMGB1 **(c) **and IL-10 **(d) **serum concentrations at the indicated times are shown for the lipopolysaccharide (LPS) (n = 6; squares) and danaparoid sodium (DA)-treated (n = 6; circles) groups. All data are expressed as mean ± SD. *Denotes a significant difference compared with the LPS group (p < 0.05).

Prior to LPS administration, IL-6, TNFα, IL-10 and HMGB1 in the serum were below levels detectable by the assays. Subsequent to LPS infusion, serum levels of IL-6 increased, with a peak value observed at 3 h in both groups. Treatment with DA following LPS administration led to a significantly decreased concentration of IL-6 at all assay times (p < 0.05) (Figure 4a). Likewise, serum levels of TNFα peaked 3 h post-LPS-infusion, with the DA treatment group showing significantly decreased levels at this time point (p < 0.05). During the investigation period, TNFα levels of DA-treated LPS group were lower than the LPS group at all assay times (Figure 4b). Serum levels of HMGB1 increased over time following LPS infusion. This increase was less prominent in DA-treated rats compared to the untreated ones. At 6, 9 and 12 h following LPS administration, HMGB1 was significantly lower in the DA-treated LPS group compared to the untreated LPS group (p < 0.05) (Figure 4c). By contrast, serum levels of IL-10 peaked 3 h post-LPS-infusion, with the DA-treatment group showing increased levels at all assay times. At 6, 9 and 12 h following LPS administration, IL-10 was significantly higher in the DA-treated LPS group compared to the untreated LPS group (p < 0.05) (Figure 4d).

#### Effect of DA on the HMGB1 levels in the lung

**Figure 5:**
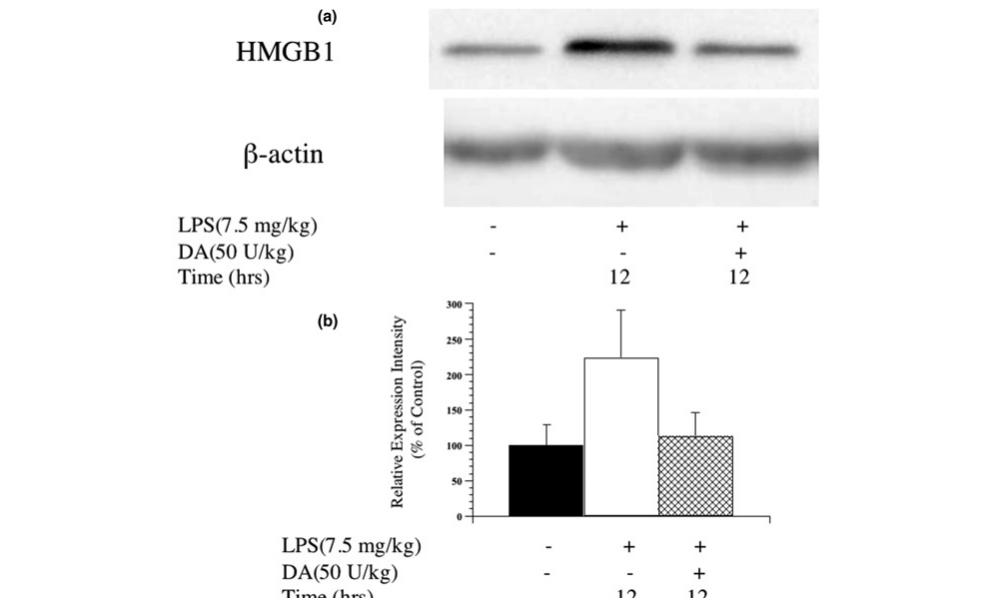
Changes in high mobility group box 1 (HMGB1) protein expression in lung tissue after lipopolysaccharide (LPS) administration in rats. **(a) **The expression of HMGB1 protein in the lung 12 h following administration of LPS in untreated LPS and danaparoid sodium (DA)-treated LPS groups was detected by Western blot. Representative blots from three separate experiments are shown. **(b) **Signal intensities for HMGB1 expression in lung tissue were quantified using an image analyser. Black bars represent the negative control group, white bars represent the LPS group, mesh bars represent the DA-treated LPS group. The expression intensity of HMGB1 protein relative to that of the negative control group was calculated for each group.

HMGB1 expression in lung tissue increased following LPS injection. This increase was less pronounced among DA-treated rats compared to the untreated LPS group (Figure 5a,b). In an immunohistochemical analysis, cells expressing HMGB1 increased following LPS administration (Additional file 1a). By contrast, the percentage of cells expressing HMGB1 decreased dramatically in the LPS-administered rats treated with DA (Additional file 1b).

### *In vitro *study

#### Effect of DA on the culture supernatant and cell protein of HMGB1

**Figure 6:**
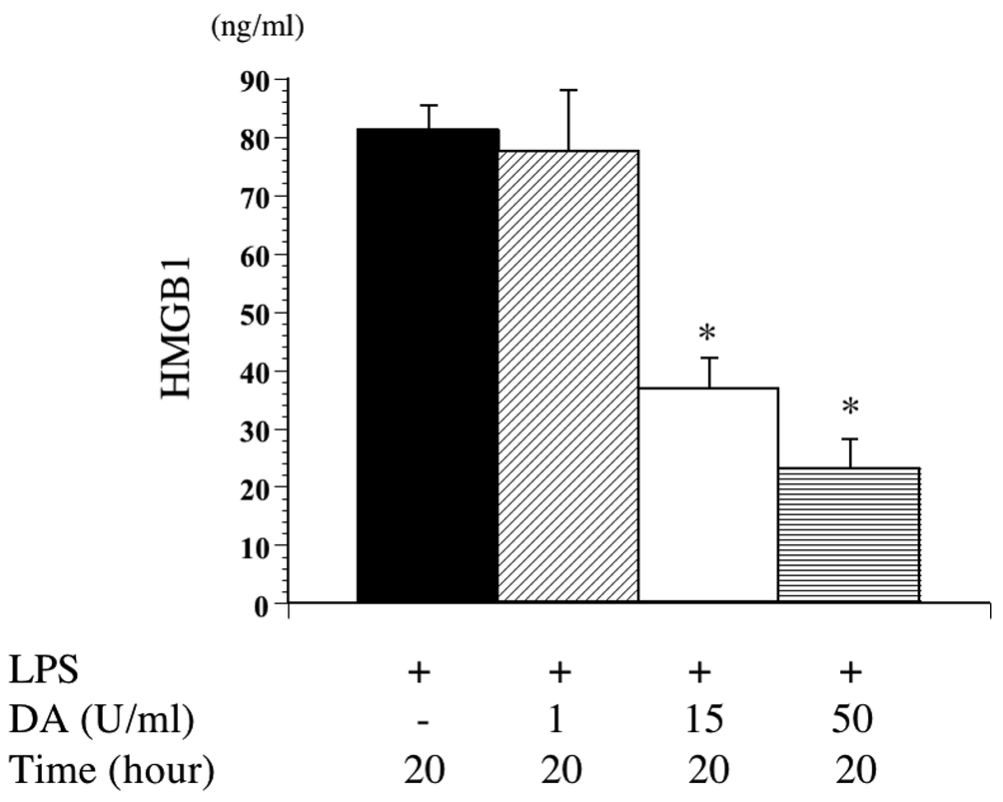
Effect of danaparoid sodium (DA) on high mobility group box 1 (HMGB1) production by lipopolysaccharide (LPS)-stimulated murine macrophages. Murine macrophages treated without or with DA (1, 15, 50 U/ml) were stimulated with LPS (100 ng/ml) for 20 h. Supernatants and cell protein were prepared and examined by enzyme linked immunosorbent assay (ELISA). All data are expressed as means ± SD. *Denotes a significant difference compared with the LPS group (p < 0.05).

The secretion of HMGB1 was measured in the culture supernatant at 20 h after the administration of LPS. The HMGB1 level of the culture supernatant increased after the administration of LPS, but the secretion of HMGB1 was inhibited by the administration of DA. In addition, the inhibition of HMGB1 by DA was minimal at a dose of 1 U/ml, was intermediate at a dose of 15 U/ml, and was maximal at a dose of 50 U/ml (Figure 6). We therefore used a concentration of 50 U/ml DA for subsequent experiments.

#### Effect of DA on the culture supernatant of cytokines

**Figure 7:**
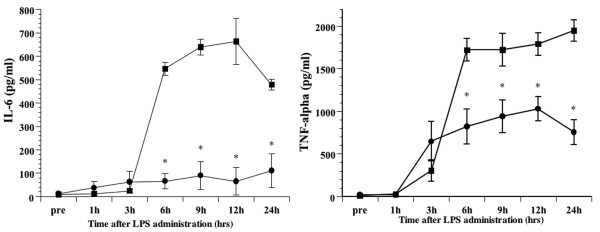
Effect of danaparoid sodium (DA) on interleukin (IL)-6 and tumour necrosis factor (TNF)α production by lipopolysaccharide (LPS)-stimulated murine macrophages. Murine macrophages treated with or without DA (50 U/ml) were stimulated with LPS (100 ng/ml) for the indicated time. Supernatants were collected and IL-6 and TNFα levels were determined by enzyme linked immunosorbent assay (ELISA). All data are expressed as mean ± SD. *Denotes a significant difference compared with the LPS-treated cells (p < 0.05).

The TNFα level in the culture supernatant increased 3 h following the administration of LPS. The administration of DA significantly inhibited the secretion of TNFα. The IL-6 level in the culture supernatant also increased after the administration of LPS. The administration of DA was thus found to significantly inhibit the secretion of IL-6 in a manner similar to TNFα (Figure 7).

#### DA inhibits the IKK pathway and modulates NF-κB

**Figure 8:**
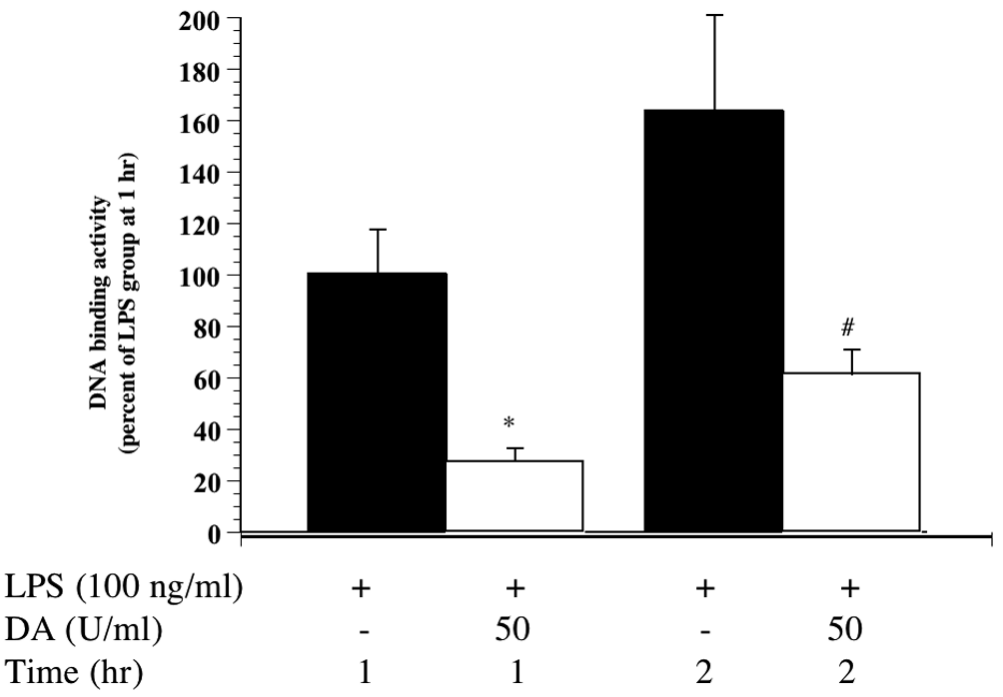
Effect of danaparoid sodium (DA) on the lipopolysaccharide (LPS)-induced increase of p50/p65 binding to DNA. The DNA binding activity assay showed a marked decrease in the p50/p65 binding activity in nuclear fractions from RAW264.7 cells. All data are expressed as the mean ± SD. *Denotes a significant difference compared with LPS group at 1 h (p < 0.05). #Denotes a significant difference compared with LPS group at 2 h (p < 0.05).

Since the NF-κB pathway plays a critical role in the secretion of cytokines, we measured the quantity of p50 and p65 in the nucleus. Treatment with LPS led to a robust activation of the NF-κB transcription factor p50/p65. This activation was partially blocked by DA (Figure 8).

We subsequently examined the IκB kinase (IKK) system as another activation agent of NF-κB. Treatment with LPS resulted in the degradation of IκB alpha and this degradation was inhibited by DA (Additional file 2). In addition, the phosphorylation of p-IκB alpha in RAW264.7 cells increased after LPS administration, and was also inhibited by DA (Additional file 2).

## Discussion

This is the first study to demonstrate the anti-inflammatory actions of DA in a rat model of endotoxin-induced lung injury. Acute inflammatory events, such as those that occur in ALI, lead to dysregulation of the coagulation cascade. Indeed, ALI is characterised by profound alterations in both systemic and intra-alveolar coagulation and fibrinolysis [17]. Activation of coagulation with resultant fibrin deposition also has proinflammatory consequences, serving to further amplify the inflammatory cascade [18]. Lung damage may result not only from the release of inflammatory mediators, but also from coagulation. These results suggest that coagulation and inflammation are related and therefore, anticoagulant therapy, such as treatment with DA, will benefit patients with ALI.

In this study, we demonstrated that treatment with the anticoagulant DA significantly improved acute lung injury and mortality in a rat model. Acute lung injury is characterised by non-cardiogenic oedema, pulmonary inflammation and severe systemic hypoxemia. Many sequelae associated with ALI result from excessive production of cytokine mediators (such as TNFα and IL-6) by activated monocytes [19]. In addition, studies have shown that HMGB1 is an important late mediator of inflammation and acute lung injury in sepsis [20-22]. This study adds to the previous findings by suggesting that DA may prevent LPS-induced lung injury by inhibiting cytokine and HMGB1 secretion.

We demonstrated that IL-10 increased following the administration of DA during endotoxin-induced systemic inflammation. A previous study showed that IL-10 inhibited the action of inflammatory cytokines [23] and had profound negative effects on macrophage activation [24]. In particular, IL-10 was closely related to the secretion of TNFα [25]. IL-10 has been identified in the lungs of patients with ARDS, where it was correlated with improved survival [26]. Based on our results, the inhibition of cytokines and prevention of lung injury might be related to increased serum levels of IL-10 resulting from administration of DA at LPS-induced systemic inflammation.

NF-κB-dependent genes are related to the development of septic shock and to septic lethality. Studies using an LPS model of septic shock have consistently demonstrated that blocking the NF-κB pathway improves outcome [27,28]. Following LPS stimulation, NF-κB is phosphorylated and coordinates the induction of several genes encoding the production and secretion of pro-inflammatory cytokines [29]. Therefore, inhibiting NF-κB activation is crucial for treating inflammation. Here, we showed that DA inhibits LPS-induced NF-κB activation, and may in turn inhibit the secretion of inflammatory mediators and improve survival rate.

Recent studies have demonstrated that coagulation, particularly the generation of thrombin, FXa, and the tissue factor-factor VIIa complex, is related to acute inflammatory responses [30]. Indeed, Riewald M *et al*., reported that FXa activates NF-κB [31]. DA is a strong inhibitor of FXa. Binding of DA to AT III leads to an accelerated inhibition of FXa, resulting in the antithrombotic effect of DA. [32]. These results suggest that the inhibitory effects of DA on NF-κB may be partially due to inhibition of FXa. Further studies are needed to clarify the signalling mechanisms that mediate the beneficial anti-inflammatory effects of DA.

Recent studies have elucidated how LPS is recognised by monocytes and macrophages of the innate immune system. LPS stimulation of murine macrophages activates several intracellular signalling pathways, including the IκB kinase (IKK)-NF-κB pathway [33,34]. We used a murine macrophage cell line to show that DA suppresses the activation of NF-κB by preventing the phosphorylation of IκB. Accordingly, the inhibition of IκB phosphorylation following DA administration in sepsis may lead to the inhibition of NF-κB activation. As a limitation of this study, the mechanisms that mediate these effects of DA in the LPS-induced systemic inflammatory model are not understood, and we need to further investigate the mechanisms of DA on the inhibition of NF-κB activation.

## Materials and methods

Antibodies to phosphorylated IkB and IkB-alpha were obtained from Cell Signaling Technology (Beverly, MA).

### Immunohistochemical analysis

Immunohistochemistry was performed after blocking endogenous peroxidase activity. Blocked sections were incubated with anti-HMGB1 polyclonal antibody (1:1000 dilution). Primary antibody binding was visualized with horseradish peroxidase conjugate and diaminobenzidine.

### Western blotting

Proteins were subjected to sodium dodecyl sulfate-polyacrylamide gel electrophoresis (SDS-PAGE), and then transferred to polyvinylidene difluoride membranes (Millipore, Bedford. MA.). The membranes were incubated with primary antibody (1:1000 dilution). After incubation with secondary antibody, blots were developed using an enhanced chemiluminescence detection kit (Amersham, Buckinghamshire, UK) and exposed on Hyperfilm ECL (Amersham, Buckinghamshire, UK).

## Conclusion

Using an LPS-induced systemic inflammation model in rats, we demonstrated that danaparoid sodium (50 U/kg) can reduce pulmonary histopathology, decrease mortality, and diminish systemic inflammatory mediators. To our knowledge, this is the first *in vivo *study that has shown such an effect. In a companion tissue culture experiment, we also demonstrated that LPS-induced secretion of cytokines can be decreased by inhibiting the IKK system. Our results suggest that DA may play a role in reducing the pathology of systemic inflammation, and that the potential mechanism of action is through the adjustment of various inflammatory mediators. Given our results, it is possible that DA may have a therapeutic effect on patients with systemic inflammation, such as septic shock, ARDS, and so on. DA has low toxicity and it is approved for the treatment of systemic inflammatory diseases.

## Key messages

Using a lipopolysaccharide (LPS) sepsis model in rats, we demonstrate that danaparoid sodium (50 U/kg) can reduce pulmonary histopathology, decrease mortality, and diminish inflammatory mediators and high mobility group box 1 (HMGB1) serum and lung levels.

In a companion tissue culture experiment, we also demonstrate that LPS-induced secretion of HMGB1 and cytokine can be decreased by inhibiting the IκB kinase (IKK) system.

Our results indicate that danaparoid sodium may play a role in reducing the pathology of sepsis, and that the potential mechanism of action is through the inhibition of systemic inflammation.

## Abbreviations

ALI = acute lung injury; ARDS = acute respiratory distress syndrome; DA = danaparoid sodium; FIIa = factor IIa; FXa = factor Xa; HMGB1 = high mobility group box 1; IKK = IκB kinase; LPS = lipopolysaccharide; NF-κB = nuclear factor κB.

## Competing interests

The authors declare that they have no competing interests.

## Authors' contributions

SH participated in the study design, performed animal, cell culture, biochemical and histological studies, and drafted the manuscript. HI planned the experimental design and performed biochemical and histological studies. SH participated in the study design and performed animal studies. SH performed animal, cell culture study and biochemical analysis. TN participated in the study design, helped to draft the manuscript and coordinated the research group. All authors read and approved the final manuscript.

## Supplementary Material

Additional file 1 Changes in the HMGB1 protein expression in lung tissue specimens after LPS administration in rats. (A) Immunohistochemcal analysis to detect HMGB1 in lung from animals killed twelve hours after 7.5 mg/kg LPS intravenous administration. The arrows indicate cells stained positive for HMGB1; ×400. (B) An immunohistochemical analysis to detect HMGB1 in the lung from animals treated with 50 units/kg DA and killed twelve hours after 20 mg/kg LPS intravenous administration; ×400. Click here for file

Additional file 2 Effect of DA on the LPS-induced phosphorylation of IkB. Murine macrophages treated with or without DA (50 units/ml) were stimulated with LPS (100 ng/ml) for 1 hr. The cytoplasmic levels of phosphorylated IkB were determined by Western blot analysis using phosphorylated IkB alpha, IkB alpha, and beta-actin antibodies. Representative blots from three separate experiments are shown. Click here for file
