# Supplementary figures and images for: Danaparoid sodium inhibits systemic inflammation and prevents endotoxin-induced acute lung injury in rats
Source: Crit Care. 2008 Apr 2;12(2):R43. doi: 10.1186/cc6851 (PMC2447588; doi:10.1186/cc6851)

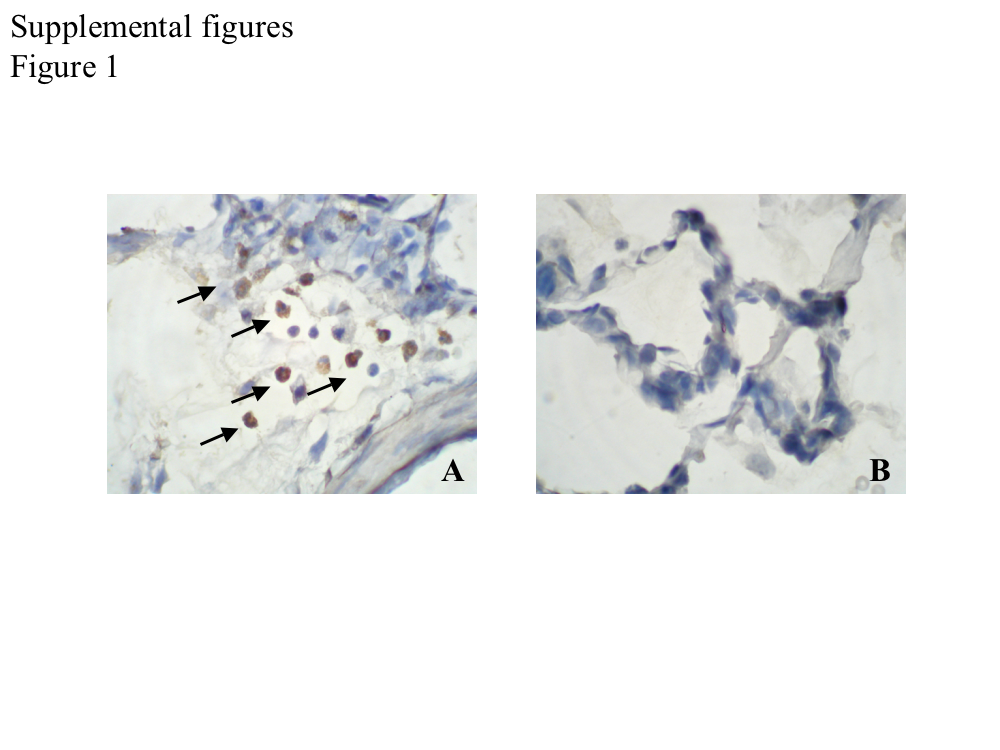

Supplement: Additional file 1 — Changes in the HMGB1 protein expression in lung tissue specimens after LPS administration in rats. (A) Immunohistochemcal analysis to detect HMGB1 in lung from animals killed twelve hours after 7.5 mg/kg LPS intravenous administration. The arrows indicate cells stained positive for HMGB1; ×400. (B) An immunohistochemical analysis to detect HMGB1 in the lung from animals treated with 50 units/kg DA and killed twelve hours after 20 mg/kg LPS intravenous administration; ×400. [file cc6851-S1.tiff]

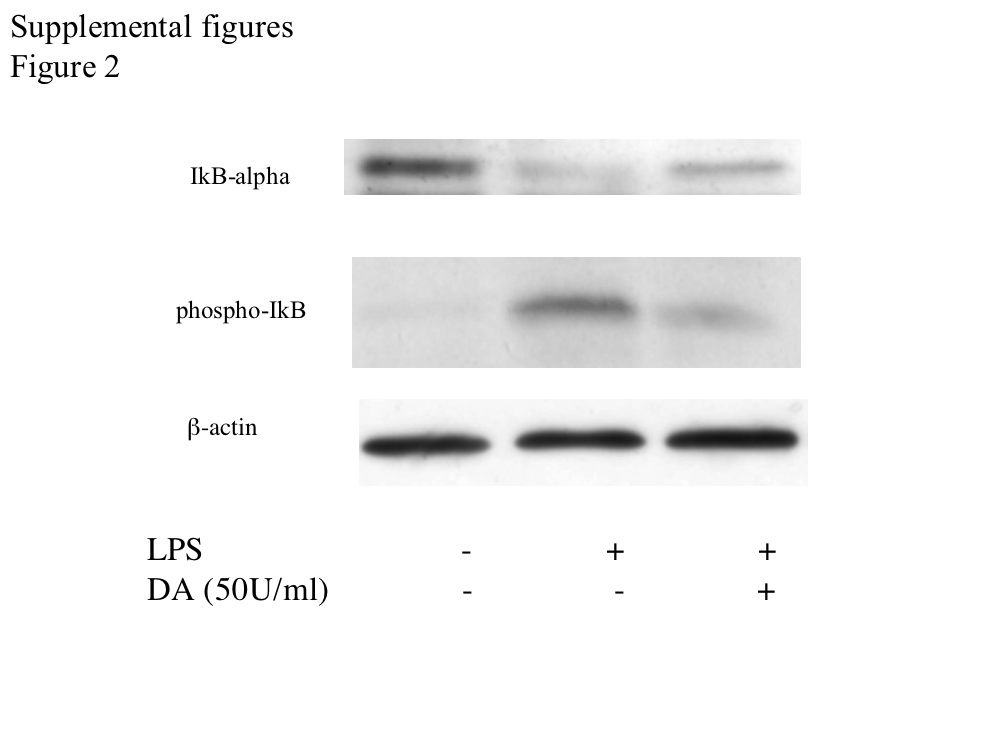

Supplement: Additional file 2 — Effect of DA on the LPS-induced phosphorylation of IkB. Murine macrophages treated with or without DA (50 units/ml) were stimulated with LPS (100 ng/ml) for 1 hr. The cytoplasmic levels of phosphorylated IkB were determined by Western blot analysis using phosphorylated IkB alpha, IkB alpha, and beta-actin antibodies. Representative blots from three separate experiments are shown. [file cc6851-S2.tiff]
